# Assessing the Performance of Chatbots on the Taiwan Psychiatry Licensing Examination Using the Rasch Model

**DOI:** 10.3390/healthcare12222305

**Published:** 2024-11-18

**Authors:** Yu Chang, Chu-Yun Su, Yi-Chun Liu

**Affiliations:** 1 Department of Psychiatry, Changhua Christian Hospital, Changhua 500, Taiwan; 182864@cch.org.tw; 2 Taichung Municipal Taichung Special Education School for The Hearing Impaired, Taichung 407, Taiwan; 3 Department of Psychiatry, Changhua Christian Children’s Hospital, Changhua 500, Taiwan

**Keywords:** chatbots, psychiatry licensing examination, Rasch model

## Abstract

Background/Objectives: The potential and limitations of chatbots in medical education and clinical decision support, particularly in specialized fields like psychiatry, remain unknown. By using the Rasch model, our study aimed to evaluate the performance of various state-of-the-art chatbots on psychiatry licensing exam questions to explore their strengths and weaknesses. Methods: We assessed the performance of 22 leading chatbots, selected based on LMArena benchmark rankings, using 100 multiple-choice questions from the 2024 Taiwan psychiatry licensing examination, a nationally standardized test required for psychiatric licensure in Taiwan. Chatbot responses were scored for correctness, and we used the Rasch model to evaluate chatbot ability. Results: Chatbots released after February 2024 passed the exam, with ChatGPT-o1-preview achieving the highest score of 85. ChatGPT-o1-preview showed a statistically significant superiority in ability (*p* < 0.001), with a 1.92 logits improvement compared to the passing threshold. It demonstrated strengths in complex psychiatric problems and ethical understanding, yet it presented limitations in up-to-date legal updates and specialized psychiatry knowledge, such as recent amendments to the Mental Health Act, psychopharmacology, and advanced neuroimaging. Conclusions: Chatbot technology could be a valuable tool for medical education and clinical decision support in psychiatry, and as technology continues to advance, these models are likely to play an increasingly integral role in psychiatric practice.

## 1. Introduction

Recent advancements in artificial intelligence (AI), particularly in the development of large language models (LLMs) employing generative AI, have led to increasingly sophisticated chatbots capable of complex interactions. These models, trained on vast amounts of text data, can generate human-like text, enabling applications in various fields, including healthcare [1,2,3,4]. For instance, Woebot is one of the most well known examples of a chatbot used in online cognitive behavioral therapy (CBT) [1]. As chatbot models continue to evolve, studies have found them increasingly capable of handling complex medical cases. For example, they have performed well on Taiwan’s Medical Licensing Examination, Japan’s Medical Licensing Examination, Medical Challenge problems, and even in specialized fields like dermatology and nephrology, with most results approaching professional standards [5,6,7,8,9,10].

The growing reliance on digital health technologies necessitates enhanced psychiatric literacy among healthcare professionals. Generative AI-powered chatbots offer a potential solution by providing readily accessible information, potentially supporting clinical decision-making and serving as educational tools [2,3]. However, psychiatry presents unique challenges for chatbots due to its complex interplay of biological, psychological, and social factors and the subjective nature of diagnosis. In a recent study, Li et al. evaluated the performance of three chatbots (ChatGPT, Bard, and Llama) in the field of psychiatric knowledge [11]. However, a knowledge gap remains. Specifically, as chatbot technology advances rapidly throughout 2024, these models have also changed significantly, necessitating a reassessment of their capabilities. Furthermore, to explore and compare their capabilities more comprehensively, the evaluation of chatbots should be expanded to include more mainstream models from different platforms.

Using standardized test questions to assess chatbot performance is an effective approach, but traditional evaluation methods may not fully capture the nuances of how these models handle questions of varying difficulty. Recognizing that traditional scoring methods may not fully capture the complexities of chatbot performance across varying question difficulties, we employed the Rasch model [12], a psychometric approach based on Item Response Theory (IRT). Unlike traditional scoring methods, the Rasch model provides a probabilistic framework for analyzing responses to test items, allowing for a better understanding of both item difficulty and respondent ability on a shared logit scale. This approach is particularly valuable for analyzing performance on standardized tests like the psychiatry licensing exam, as it allows for a direct comparison of chatbot abilities across varying question difficulties. This enables a more precise identification of specific areas of strength and weakness [13]. While previous studies have evaluated chatbot performance on medical exams, the application of the Rasch model in this context is relatively novel. This may be due to the recent emergence of highly capable chatbots and the growing need for more sophisticated evaluation methods that go beyond simple accuracy metrics.

To fill these knowledge gaps, our study aimed to systematically evaluate and compare the performance of several state-of-the-art chatbots on the most recent 2024 Taiwan psychiatry licensing examination. We utilized the Rasch model to compare different versions of mainstream chatbots, with a particular focus on the performance of the top-performing model. Further analysis of the top-performing model could provide valuable insights into how it handles different types of questions.

## 2. Materials and Methods

We utilized all 100 psychiatry multiple-choice questions, each with five options and one correct answer, from the 2024 Taiwan psychiatric licensing examination. This examination, administered in traditional Mandarin by the Taiwanese Society of Psychiatry (TSP), is a nationally standardized test required for psychiatric licensure in Taiwan. It assesses the core knowledge and clinical reasoning skills expected of practicing psychiatrists. Each question ranged from approximately 20 to 700 Mandarin characters in length, with longer questions often presenting detailed clinical scenarios. The questions represent the six key domains assessed in the exam: Pathophysiology and Epidemiology (30 questions); Diagnostic Assessment and Clinical Examination (23 questions); Psychopharmacology and Other Therapeutic Modalities (19 questions); Psychosocial and Cultural Influences (4 questions); Neuroscience and Behavioral Science (12 questions); and Forensic Psychiatry and Ethics (12 questions). Using the complete exam ensures a representative assessment of chatbot performance across the full spectrum of knowledge required for licensure and provides a standardized benchmark against which to evaluate their capabilities. Furthermore, as current chatbot evaluation relies heavily on text-based input and output, the psychiatry licensing examination, which assesses knowledge and reasoning through textual questions, offers a valid and relevant method for gauging chatbot abilities in this context. Each question was worth one point, with a passing score of 60, established by expert consensus. All questions were carefully crafted by experienced psychiatric specialists in Taiwan to ensure both relevance and accuracy.

The chatbots included in this study were selected based on their performance rankings on the LMArena benchmark [14]. LMArena provides a comprehensive evaluation of various state-of-the-art language models, ranking them based on multiple criteria, including their ability to handle complex tasks and their overall performance across different domains. We first identified the top-performing models on the LMArena overall leaderboard. Then, we examined the platforms (e.g., OpenAI, Anthropic, Google, Meta, and Mistral) that developed these leading models. Finally, from these platforms, we selected all available chatbot models. This process ensured that our study focused on a diverse set of leading chatbots representing the current state of the art in chatbot technology. The specific models included in our analysis were OpenAI’s ChatGPT (including UI-accessible versions like 4o, 4o-mini, o1-preview, and o1-mini, and API-accessible models like 4-Turbo and 4o-latest), Anthropic’s Claude (including versions 2.1, 3-Opus, 3-Sonnet, and 3-Haiku), Google’s Gemini (including versions 1.0pro, 1.5pro, and experimental versions of 1.5pro), xAI’s Grok (including versions 2 and 2-mini), Meta’s Llama (including versions 3.1-405B and 70B), and Mistral AI (version 2-large).

We conducted the testing process between 10 September 2024 and 12 September 2024. The testing process consisted of presenting each chatbot with 100 psychiatry exam questions in turn, with 10 questions per batch. For each batch, the following prompt was provided in traditional Mandarin: “The following are single-choice questions, each with one correct answer. Please provide the correct answer for the following ten questions.” Due to the context length limitations of some chatbots, we decided to ask 10 questions per batch to ensure a consistent prompt format across all models. The chatbots were required to answer all 100 questions in this manner, with no prior knowledge of the test content or any additional context. The responses were recorded for each question. The primary outcome of this study was chatbot performance on the 2024 Taiwan psychiatry licensing examination, measured as chatbot ability estimated in logits using the Rasch model. Secondary outcomes included the following: (1) a passing score analysis, examining chatbots achieving a passing score (≥60) on the exam and how this performance has changed over time based on chatbot release dates; and (2) an answering process analysis of the top-performing chatbot, focusing on its responses to the easiest items answered incorrectly and the most difficult items answered correctly to identify specific strengths and weaknesses in its reasoning.

### Statistical Analysis

First, we calculated each chatbot’s score using traditional methods and summarized the results using the mean, standard deviation, maximum and minimum scores, and Cronbach’s alpha. Before applying the Rasch model, we conducted preliminary analyses to ensure the data met the assumption of unidimensionality [15], meaning that the test measures a single underlying trait—psychiatric knowledge. This ensures that all the questions in the test consistently assess the chatbots’ understanding and application of psychiatric concepts, without introducing additional unrelated dimensions. A principal component analysis (PCA) of the residuals was performed, with a variance explained by the Rasch dimension above 20% considered acceptable [15]. After confirming that the data met the fundamental assumptions of the Rasch model, we proceeded with the following analyses: (1) estimation of item difficulty and chatbot ability parameters using Joint Maximum Likelihood Estimation (JMLE); (2) assessment of model fit using infit and outfit mean square (MNSQ) statistics; and (3) visualization of the results using a person–item map (PKMAP) [16].

We used JMLE to estimate the ability of each chatbot and the difficulty of each test item. This method computes the likelihood of a correct response based on both the chatbot’s ability and the item’s difficulty. It is an iterative process that finds the values of these parameters that maximize the likelihood of observing the actual response patterns in the data. This is achieved by repeatedly refining the estimates until the likelihood function converges to a maximum value. The standard error (SE) of the JMLE estimates is calculated as the square root of the variance of the estimates, providing a measure of the uncertainty associated with each estimate. The probability P that a chatbot *n* will answer a question *i* correctly is given by the logistic function [17]:

(1) $$P\left(X_{ni}=1\right)=\frac{e^{\theta n-\delta i}}{1+e^{\theta n-\delta i}}$$

This equation implies that when the chatbot’s ability *θn* matches the difficulty of the question *δi*, the probability of answering the question correctly is 0.5 (i.e., a 50% chance). As ability *θn* increases relative to item difficulty *δi*, the probability of a correct response increases. We used the Wald test to evaluate whether the observed differences in JMLE estimates between chatbots were statistically significant. A *p*-value of less than 0.05 indicated statistical significance.

We calculated both infit and outfit mean square statistics to assess the fit of the chatbot’s performance to the Rasch model’s expectations [13]. These statistics compare the observed response patterns of each chatbot with the expected patterns based on the estimated item difficulties and chatbot abilities. The infit MNSQ is calculated by weighting the squared standardized residuals using the expected variance, making it more sensitive to inconsistencies in responses to items near the chatbot’s ability level. The outfit MNSQ, on the other hand, weights the squared standardized residuals using the observed variance, making it more sensitive to unexpected responses to very easy or very difficult items. Values between 0.5 and 1.5 for both infit and outfit MNSQ are generally considered acceptable, indicating adequate fit to the Rasch model [18].

A person–item map was generated for each chatbot, particularly for the best-performing model, to visually represent how well the chatbot performed across questions of varying difficulty. This map helped identify specific strengths and weaknesses by showing which questions were answered correctly or incorrectly relative to their difficulty level. To further investigate the responses of the top-performing chatbot, we analyzed its explanations for the easiest items answered incorrectly and the hardest items answered correctly. For each of these selected items, the chatbot was prompted with the following question in traditional Mandarin: “Can you explain the reasoning behind your answer to this question?” These explanations, along with the original questions, correct answers, and the chatbot’s initial responses, formed the basis for the answering process analysis described below.

All statistical analyses were conducted using WINSTEPS (version 5.8.1) software on the Windows operating system. WINSTEPS is a leading commercial software for Rasch analysis due to its robust JMLE implementation and comprehensive fit statistic.

## 3. Results

### 3.1. Passing Score Analysis

We collected the answering results from a total of 22 chatbots (Table 1). The average raw score was 60.4, with a sampled standard deviation of 12.0, a maximum raw score of 85, and a minimum raw score of 38. The Cronbach’s alpha for the test’s person score reliability was 0.89. The table (Table 2) below summarizes the test results of the chatbots along with their release dates. Many leading chatbots released in February 2024 onward achieved the passing score of 60 points on the psychiatry licensing examination, which marks a significant advancement in the application of chatbot knowledge. For instance, Gemini-1.5pro, released in February 2024, scored 62 points, while ChatGPT-o1-preview achieved an impressive 85 points in the test conducted on 12 September 2024, far exceeding the standard.

### 3.2. Unidimensionality

The analysis showed that the raw variance explained by the measures was 35%. This value represents the proportion of total variance in the data accounted for by the principal component analysis of residuals, indicating the degree to which a single latent trait (psychiatric knowledge) explains the observed response patterns. A value exceeding the commonly accepted 20% threshold [15], as observed in our study, supports the unidimensionality of the data.

### 3.3. Rasch Model Analysis

ChatGPT-o1-preview achieved a JMLE estimate of 2.36 (Table 1), the highest among all participating chatbots, demonstrating its superior performance on more difficult questions. Its infit mean square was 1.09, which was within the acceptable range of 0.5 to 1.5, indicating consistency with model expectations. Additionally, its outfit mean square was 0.63, which also falls within the acceptable range. These fit statistics being within the acceptable range indicates that ChatGPT-o1-preview’s responses are largely consistent with the Rasch model’s expectations, enhancing the reliability of the ability estimate and supporting the validity of applying the Rasch model to this dataset. The JMLE estimate for the passing score of 60 points was 0.44. ChatGPT-o1-preview showed a statistically significant superiority in ability (*p* < 0.001), with a 1.92 logits improvement compared to the passing threshold. This difference translates to a substantially higher probability of answering questions correctly (e.g., approximately 87% probability compared to 50% at the threshold).

### 3.4. Person–Item Map (PKMAP) Chart for ChatGPT-o1-Preview

**Figure 1:**
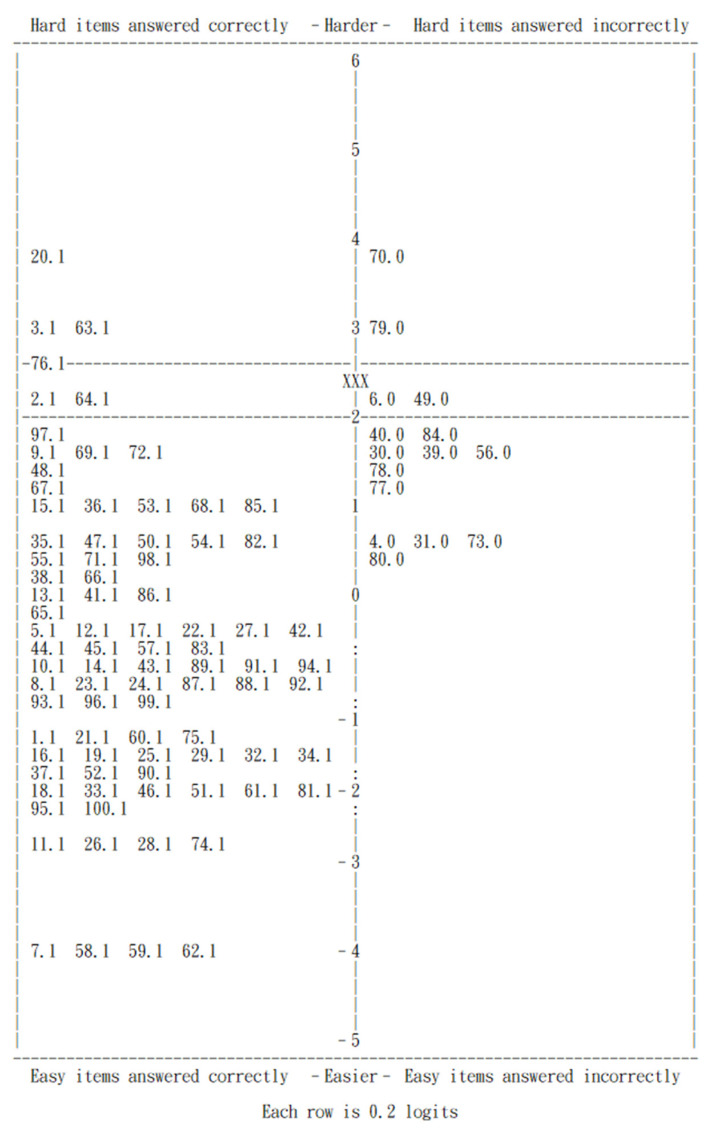
Person–item map (PKMAP) of ChatGPT-o1-preview. Vertical units in the map represent logits. The mark “XXX” indicates the chatbot’s ability level. Each item in the map corresponds to a question number from the examination, with a “1” or “0” placed after the item number. A “1” indicates that the question was answered correctly and is positioned on the left side of the map, while a “0” indicates that the question was answered incorrectly and is positioned on the right side. The difficulty of each item is also represented by its position along the vertical axis, showing how challenging the question was relative to the chatbot’s ability.

The following PKMAP (Figure 1) chart illustrates ChatGPT-o1-preview’s performance on questions of varying difficulty. Vertical units represent logits, and “XXX” marks the chatbot’s ability level. Each item corresponds to a question number from the exam. The difficulty of each item is also represented by its position along the vertical axis. The lower-right section of the PKMAP shows easier questions (e.g., Items 4, 31, 73, 77, 78, and 80) that the chatbot answered incorrectly, despite being below the difficulty threshold where a proficient model would typically succeed. In contrast, the upper-left section of the PKMAP highlights more challenging questions (e.g., Items 3, 20, and 63) that the chatbot answered correctly, demonstrating its ability to successfully tackle complex psychiatric problems.

### 3.5. Answering Process Analysis

The answering process analysis focused on the easiest items answered incorrectly and the hardest items answered correctly by ChatGPT-o1-preview, as identified by the PKMAP (Figure 1). We examined the chatbot’s responses and the explanations it provided when prompted (as described in the Statistical Analysis section). Based on this analysis, we identified key strengths and weaknesses in the chatbot’s reasoning, which are summarized and illustrated in Table 3. ChatGPT-o1-preview excelled on more difficult questions, particularly those requiring ethical judgment and conceptual understanding. However, it struggled with easier questions, often due to challenges related to legal knowledge, adapting to recent or regional contexts, and some specialized psychiatric knowledge, such as psychopharmacology and advanced neuroimaging.

## 4. Discussion

To the best of our knowledge, this is the first study to comprehensively evaluate the performance of multiple chatbots in the field of psychiatry using the Rasch model. This approach allowed us to conduct an in-depth analysis of the chatbots’ abilities, providing insights beyond simple accuracy metrics. The results of the passing score analysis highlight the rapid advancements in chatbot capabilities, with several models released in February 2024 onward surpassing the passing score of 60. Notably, in the Rasch model analysis, ChatGPT-o1-preview achieved the highest score of 85 and the highest JMLE estimation of 2.36, demonstrating its advanced capacity to solve complex psychiatric problems. For instance, compared to a chatbot scoring 60 with a 50% probability of answering a question correctly, ChatGPT-o1-preview showed a superior ability of 1.92 logits, which corresponds to an 87% probability of answering correctly. However, despite its overall strong performance, the answering process analysis revealed that even top-performing models still face limitations, particularly when it comes to interpreting recent legal amendments, handling region-specific regulations, and navigating specialized medical knowledge.

Our findings aligned with previous research, such as the study by Li et al. [11], which demonstrated that earlier versions of ChatGPT had already passed the exam and outperformed models like Llama and Bard. The results of our study showed that more models, including GPT, have now passed the exam with significantly improved performance, and GPT-based models continue to outperform other models at the time of assessment. This aligned with OpenAI’s recent emphasis on enhancing these models’ deductive reasoning and critical judgment capabilities, particularly through techniques like Chain of Thought (CoT) [19,20]. CoT allows the model to break down complex problems step by step, leading to more accurate interpretations of the questions posed in our study. This might explain why ChatGPT-o1-preview was able to achieve superior accuracy in handling intricate psychiatric questions.

The strong performance of chatbots on the psychiatry licensing examination, particularly when analyzed using the Rasch model, highlights their potential to revolutionize digital health and AI-based examination tools. By achieving scores that exceed the passing threshold and demonstrating advanced reasoning capabilities, these chatbots could serve as powerful tools in medical education and clinical decision support. For instance, chatbots can provide personalized learning experiences by simulating clinical scenarios, offering instant feedback on diagnostic decisions, and adapting to the learner’s performance to focus on areas that need improvement. This makes them ideal for self-paced learning and competency-based assessments. In clinical settings, chatbots could provide differential diagnoses for a patient presenting with mixed anxiety and depression symptoms, suggest treatment guidelines for schizophrenia, or offer a second opinion on managing a bipolar disorder manic episode. This can reduce cognitive load and support clinicians in staying up to date with best practices. Similar findings from other studies provide explanations and evidence of chatbots’ applicability in medical education and clinical decision support [2,3,21,22]. However, further research is needed to test these models in real-world applications, ensuring that they can perform effectively outside of controlled environments.

Chatbots still face dilemmas, particularly in interpreting recent legal amendments or region-specific regulations. These types of information are often not included in training data and, in many cases, cannot be inferred from general knowledge. Additionally, gaps in specialized medical knowledge were also noted. In clinical settings, these limitations may lead to incorrect legal advice or suboptimal treatment recommendations. From an educational perspective, the use of chatbots might result in learners developing incomplete or incorrect knowledge.

To address these dilemmas, three potential strategies stand out: fine-tuning, prompt engineering, and retrieval-augmented generation (RAG) [23,24,25,26]. Fine-tuning involves retraining the model with additional, domain-specific data, allowing it to better handle specialized knowledge areas. Prompt engineering involves crafting prompts in a way that maximizes the model’s output accuracy, potentially incorporating tailored data to guide the model’s responses more effectively. RAG allows the chatbot to retrieve relevant information from external databases, enhancing its ability to provide accurate, up-to-date responses. However, all three strategies require close collaboration with psychiatrists, to ensure that the training content is not only relevant but also of high quality. While current models are built as general-purpose models, integrating specialized psychiatric knowledge requires careful selection of the most appropriate and reliable information. Therefore, the involvement of psychiatrists is essential to guide this process. Given the rapid pace of development in both psychiatry and chatbot technology, it will be increasingly important for psychiatrists to play an active role in shaping the future of chatbots in the field.

Ethical considerations are also critical when integrating chatbots into psychiatry. Issues such as privacy, data security, and accountability must be addressed, especially given the sensitive nature of psychiatric patient data [27]. It is essential that AI systems comply with privacy regulations and securely store and process patient information. For instance, chatbots should anonymize patient data and protect them from unauthorized access. Another key issue is accountability: if a chatbot provides incorrect treatment recommendations or misinterprets legal guidelines, who is responsible—the clinician, the AI developer, or the healthcare institution? Clear guidelines are needed to delineate the responsibilities of all stakeholders involved in the use of chatbots in clinical settings. Our study found that advanced chatbots are capable of ethical reasoning, suggesting their potential to assist clinicians in making ethical decisions. However, AI systems should always operate under appropriate human supervision to ensure that patient autonomy and clinical judgment remain central to the decision-making process. [2,27,28].

The introduction of chatbots into psychiatry could significantly impact the doctor–patient relationship. On one hand, chatbots can reduce clinician cognitive load by automating routine tasks, such as collecting patient history or generating differential diagnoses, allowing clinicians to focus more on meaningful interactions with patients. For instance, when chatbots handle administrative tasks, clinicians can dedicate more time to building therapeutic rapport. On the other hand, an over-reliance on chatbots may undermine the therapeutic alliance if patients feel their care is driven by a machine rather than a human expert. In psychiatry, where trust and rapport are crucial, this could have negative consequences. Therefore, it is essential to ensure that chatbots are used as complementary tools, augmenting but not replacing the clinician’s role in patient care. Additionally, chatbots in psychiatry could help reduce the stigma surrounding mental health treatment by offering anonymous and accessible support, encouraging more individuals to seek help [27].

Our study had several limitations. First, it focused on performance in a controlled environment using 100 psychiatry licensing exam questions from a specific country. While these questions are designed to evaluate clinical knowledge, they may not fully present the complexity of real-world psychiatric practice, especially in dynamic, complex situations involving patient and caregiver interactions, multidisciplinary collaboration, and cultural considerations [29]. Second, the study was limited to a specific set of chatbots and may not fully represent the performance of all available models on the market. As chatbot technology rapidly advances, new models with differing architectures, training data, and capabilities are continually being developed. Finally, there is an inherent bias in chatbot training data. Since these models are trained on large datasets that may not fully reflect the diversity of psychiatric cases across populations, their recommendations could be skewed toward certain demographic groups or clinical approaches [29].

## 5. Conclusions

Our study provides statistically significant evidence of the increasing capabilities of generative AI in professional licensing, as demonstrated by chatbot performance on the psychiatry licensing exam. These findings suggest the potential of these tools to enhance medical education and assist clinical decision-making. As chatbot technology continues to evolve, the involvement of psychiatrists will be critical in ensuring these systems are optimized for practical use. Future work should focus on testing these models in real-world settings and developing legal and ethical frameworks to support their responsible and complementary use in psychiatry.

## Figures and Tables

**Table 1:** Chatbots’ raw scores and parameters in Rasch analysis.

Model Name	Raw Score	JMLE	JMLE SE	Infit MNSQ	Outfit MNSQ
ChatGPT-o1-preview	85	2.36	0.33	1.0906	0.6257
Grok-2	78	1.71	0.29	1.0292	0.7259
ChatGPT-4o-latest	76	1.55	0.28	0.812	0.6417
Claude-3.5-Sonnet	71	1.17	0.27	0.8714	0.7327
Gemini-1.5pro-Exp0827	70	1.1	0.27	0.8727	0.8008
Llama-3.1-405B	69	1.03	0.26	0.727	0.5884
Grok-2-mini	67	0.89	0.26	0.8102	0.716
Gemini-1.5Flash-Exp0827	63	0.63	0.25	0.8978	0.7194
ChatGPT-4o	63	0.63	0.25	1.3241	1.7568
Claude-3-Opus	63	0.63	0.25	0.9868	1.0439
Gemini-1.5pro	62	0.57	0.25	0.9765	0.8139
ChatGPT-4-Turbo	62	0.57	0.25	0.9757	0.9276
Gemini-1.5Flash	60	0.44	0.25	0.7196	0.5758
Claude-3-Haiku	57	0.25	0.25	0.9862	1.09
Claude-3-Sonnet	54	0.07	0.25	0.8871	0.8839
ChatGPT-o1-mini	53	0.01	0.25	1.1565	1.377
Llama-3.1-70B	53	0.01	0.25	1.2604	1.3105
Gemini-1.5Flash-8B-Exp0827	47	−0.35	0.25	0.9615	0.9993
Claude-2.1	47	−0.35	0.25	1.1472	1.1165
Mistral-large-2	47	−0.35	0.25	1.2416	1.2356
ChatGPT-4o-mini	43	−0.6	0.25	1.1073	1.0592
Gemini-1.0pro	38	−0.91	0.25	1.1394	1.8598

JMLE, Joint Maximum Likelihood Estimation; SE, standard error; MNSQ, mean square.

**Table 2:** Chatbots’ raw score sorted by ascending release date.

Model Name	Raw Score	Release Date
Gemini-1.0pro	38	2023/12/6
Gemini-1.5pro	62	2024/2/15
Claude-3-Opus	63	2024/3/14
Claude-3-Sonnet	54	2024/3/14
ChatGPT-4o	63	2024/5/13
Claude-3.5-Sonnet	71	2024/6/20
Llama-3.1-405B	69	2024/7/23
ChatGPT-4o-latest	76	2024/8/8
Grok-2	78	2024/8/13
Gemini-1.5pro-Exp0827	70	2024/8/27
ChatGPT-o1-preview	85	2024/9/12

**Table 3:** Strengths and weaknesses of answering process analysis.

Strengths: Hard Items Answered Correctly		
No.	Answering Process	Interpretation
20	The chatbot correctly identified that “actively participating in eliminating discrimination in healthcare” aligns with the principle of social justice, rather than patient welfare.	Precise ethical understanding
3	The chatbot correctly identified that qualities such as loyalty and commitment, while important personal traits, are not considered independent developmental tasks.	Precise conceptual understanding
63	This question referenced a “newly revised” Mental Health Act. The model excluded an option to avoid assuming potentially incorrect legal information and instead relied on general legal and clinical practices regarding minors (e.g., requiring guardian involvement for prescriptions and counseling).	Precise ethical understanding Ability to deduce Awareness of Knowledge Limits
Weakness: Easy Items Answered Incorrectly		
80	According to Taiwanese law, individuals with an IQ below 85 may be exempt from service, but the model failed to identify this threshold correctly.	Struggle with adapting to localized regulatory contexts
4	The chatbot incorrectly stated that the term for psychiatrists serving as jurors is 2 years, whereas the correct term is 3 years.	Difficulty in handling specific legal knowledge
31	The chatbot hesitated to confirm the accuracy of the correct interpretation of PET imaging results in generalized anxiety disorder patients.	Lack of confidence when dealing with advanced neuroimaging knowledge
73	An incorrect response was provided when asked about the effective date of the amendment to the Criminal Procedure Law, which was promulgated on 15 December 2023, with a 5-month delay before coming into effect.	Struggle with time-sensitive legal information
77	The chatbot incorrectly suggested that SSRIs frequently cause a reduction in platelet count, whereas the more common side effect is reduced platelet aggregation, not a decrease in the number of platelets.	Limitations in specialized medical knowledge in pharmacological effects
78	The chatbot correctly identified that herpes simplex encephalitis (HSE) primarily affects the temporal and frontal lobes, but downplayed the prominence of olfactory hallucinations as a key psychiatric symptom.	Struggle with specialized medical knowledge in psychopathology

## Data Availability

Restrictions apply to the availability of these data. Data were obtained from the Taiwanese Society of Psychiatry and are available at https://www.sop.org.tw/news/n_list.asp (accessed on 9 September 2024).
